# 

**Published:** 1893-05-13

**Authors:** 


### CONTENTS.

Why do Doctors not Advertise? - - - - - - - 97

Around the Hospitals - - - - - - - - - - - 98

Medical History from the Earliest Times. By E. T. Withington, M.A., M.B Oxon. LIV.—Van Helmont - - 99

Contemporary Theory and Research - - - - - - 100

How to Keep Well in Africa. I.—Prevention - - - - 101

Gleanings in Science and Medicine - - - - - - 102

Notes and News - - - - - - - - - - - - 104

The Hospital Clinic—Leeds General Infirmary.—The Surgical Treatment of Gallstones - - - - - - - - - 105

The London Hospital.—The Treatment of Abscesses - - 106

Rhinoscopy - - - - - - - - - - - - 107

New Drugs, Appliances, and Things Medical.—Malto-Pepsine 108

Annotations.—Milk in Bright's Disease—Medical Practice in Australia—Physiological Pessimism - - - - - - - 103

The Institutional Workshop—

Within the Hospitals.—Three Cottage Hospitals - - - 109

Practical Departments.—L.K. Batteries - - - - - - 109

The Story of the Insane from Year to Year - - 110

Festival Dinners and Meetings.—The North-Eastern Hospital for Children; the Orphan Working School, Haverstock Hill; British Home for Incurables, Clapham - - - 111

Construction Notes - - - - - - - - - - 112

Scraps and Gleanings - - - - - - - - - - 112

The Book World of Medicine and Science - - - - vii

Medical Vacancies and Appointments - - - - - - ix

### EXTRA SUPPLEMENT.—THE NURSING MIRROR.

News from the Nursing World.—Miners at Windsor—Defined Duties—The Invalid Children's Aid Association—The Nurse Dolls—Rules—Dangerous Playgrounds—Voluntary Help—Unreasonable Objections—Parisian Precocity—A Convalescent in Gaol—Lepers in New South Wales—The Disabled Nurse—"The Hospital" Endowed Bed - - - lxi

Sanitation for Nurses. By A Sanitary Inspector. V.—Notification in Foreign Countries. Italy - - - - - lxiii

Pensions for Nurses - - - - - - - - - - - lxiv

Minor Appointments—Wants and Workers - - - lxiv

A German Hospital. By An English Patient. III.—Daily Life in the Ward - - - - - - - - - - lxv

Warnings - - - - - - - - - - - - - - lxvi

Appointments - - - - - - - - - - - - - lxvi

Hints for Nurses.—Poultices for Private Patients - - - lxvi

Where to Go—Notes and Queries - - - - - - - lxvi

Queen Victoria Jubilee Institute.—Lecture on Nursing in Cholera. By Sir Joseph Fayrer, K.C.S.I., M.D. - - lxvii

The Muses' Looking Glass. Pictures.—The New Gallery lxviii

The Book World for Women and Nurses - - - - lxix

